# Seawater acidification induced immune function changes of haemocytes in *Mytilus edulis*: a comparative study of CO_2_ and HCl enrichment

**DOI:** 10.1038/srep41488

**Published:** 2017-02-06

**Authors:** Tianli Sun, Xuexi Tang, Yongshun Jiang, You Wang

**Affiliations:** 1 Department of Marine Ecology, College of Marine Life Sciences, Ocean University of China, Qingdao 266003, China

## Abstract

The present study was performed to evaluate the effects of CO_2_− or HCl-induced seawater acidification (pH 7.7 or 7.1; control: pH 8.1) on haemocytes of *Mytilus edulis*, and the changes in the structure and immune function were investigated during a 21-day experiment. The results demonstrated that seawater acidification had little effect on the cellular mortality and granulocyte proportion but damaged the granulocyte ultrastructure. Phagocytosis of haemocytes was also significantly inhibited in a clearly concentration-dependent manner, demonstrating that the immune function was affected. Moreover, ROS production was significantly induced in both CO_2_ and HCl treatments, and four antioxidant components, GSH, GST, GR and GPx, had active responses to the acidification stress. Comparatively, CO_2_ had more severe destructive effects on haemocytes than HCl at the same pH level, indicating that CO_2_ stressed cells in other ways beyond the increasing H^+^ concentration. One possible explanation was that seawater acidification induced ROS overproduction, which damaged the ultrastructure of haemocytes and decreased phagocytosis.

Ongoing ocean acidification and related changes in ocean carbonate chemistry will contribute to major changes in marine ecosystems^1^. Due to the slow vertical mixing of seawater, excessive H^+^ is retained at the surface for a prolonged period of time. Therefore, an approximately 0.4-unit decrease in the pH is predicted to occur by the year 2100^2^. To cope with this crisis, almost 40 million tons of CO_2_ have been legally captured and stored per annum in sub-seabed geological formations^3^. However, 0.2% (approximately 80 thousand tons) of the total gas storage was estimated to escape into the seawater^4^. Therefore, reasonable assessments or predictions of the potential ecological impacts of both near-future and extreme scenarios of seawater acidification on the key marine organisms are of great importance. Regarding seawater acidification, a growing body of evidence supports that the elevation of CO_2_ levels has a strong impact on the acid-base balance, the energy metabolism and the biomineralization of marine organisms, especially of calcifying organisms such as bivalves^5^,^6^. Previous studies proposed that invertebrate bivalve molluscs would be more sensitive to ocean acidification stress than highly mobile organisms such as marine fishes^7^,^8^. Barton *et al*.^9^ posited that massive die-offs in the wild oyster populations and hatcheries along the US west coast are due to the upwelling of acidic waters, which is exacerbated by ongoing ocean acidification^9^. Our previous study also showed that seawater acidification had a negative impact on the physiological processes of *Mytilus edulis*, inhibited their metabolic activities and carbon sink ability, and significantly increased their mortality level^10^.

The ability of marine organisms to adapt to acidified conditions will be critical to their health and ultimate survival. The immune strategy of bivalves is merely based on an innate, non-lymphoid immune system comprising haemocytes and soluble haemolymph factors, which differs from the immune system reported in vertebrates^11^. Phagocytosis by circulating haemocytes is one of the major internal defences in the bivalve immune response and is followed by the release of reactive oxygen species (ROS) metabolites and degradative enzymes, as well as the secretion of cytotoxic molecules^12^. However, bivalves are considered poor regulators of the haemolymph acid-base balance^13^,^14^. Li *et al*.^15^ showed that ocean acidification (−0.3 and −0.6 pH units) decreased the haemolymph pH value of *Pinctada fucata* by 0.45–0.55 pH units^15^. Michaelidis *et al*.^13^ demonstrated that a seawater pH below 7.5 would cause permanent reductions in the haemolymph pH of *M*. *galloprovincialis*^13^. Decreases in pH have significant effects on bivalve health. Matozzo *et al*.^16^ demonstrated that seawater acidification significantly affected the immune parameters in two species of bivalves, *M*. *galloprovincialis* and *Chamelea gallina*^16^. Bibby *et al*.^17^ found that CO_2_-induced acidification (−0.2 to −1.1 pH units) had an obvious impact on the physiological condition and functionality of *M*. *edulis* haemocytes, and their phagocytosis was strongly decreased with decreasing pH levels^17^. Li *et al*.^15^ found that acidification changed the community structure of *P*. *fucata* haemocytes and that the percentages of large hyalinocytes and granulocytes increased while the neutral red uptake ability decreased^15^. However, there are few studies on the overall effects of elevated oceanic CO_2_ on haemocytes from *M*. *edulis*, especially in terms of the toxic mechanism of acidification in different biospectra of haemocytes and their immune function.

We conducted a 21-day experiment to investigate the impact of CO_2_ enrichment-induced seawater acidification on key aspects of the haemocyte structure and immune function of *M*. *edulis*. Mussels were exposed to pH levels mimicking near future ocean acidification (pH 7.7) or CO_2_ leakage scenarios (pH 7.1). Considering that more complex effects beyond acidification would occur during the dissolution of CO_2_ in seawater, we also applied mineral acid (HCl)-induced seawater acidification for comparison^18^. The results in the present study shed light on how seawater acidification contributes to the structure and function of the haemocytes of *M*. *edulis*.

## Results

### Effects of seawater acidification on different biospectra of haemocytes

The percentages of haemocytes that were found to be nonviable were very low in the control (2.8 ± 0.37%) and acidified groups (7.7 HG: 4.1 ± 0.42%; 7.7 CG: 4.5 ± 0.48%; 7.1 HG: 3.9 ± 0.52%; 7.1 CG: 4.0 ± 0.34%;). Although the average mortality of haemocytes increased in all acidified groups, the changes were not statistically significant (P > 0.05). Meanwhile, the fraction of granulocytes in both the treatment and control groups remained at 44.8 ± 7.0% of the haemocyte population over the 21-d period. Therefore, acidification did not have a significant effect on the percentage of granulocytes in the total haemocytes.

**Figure 1:**
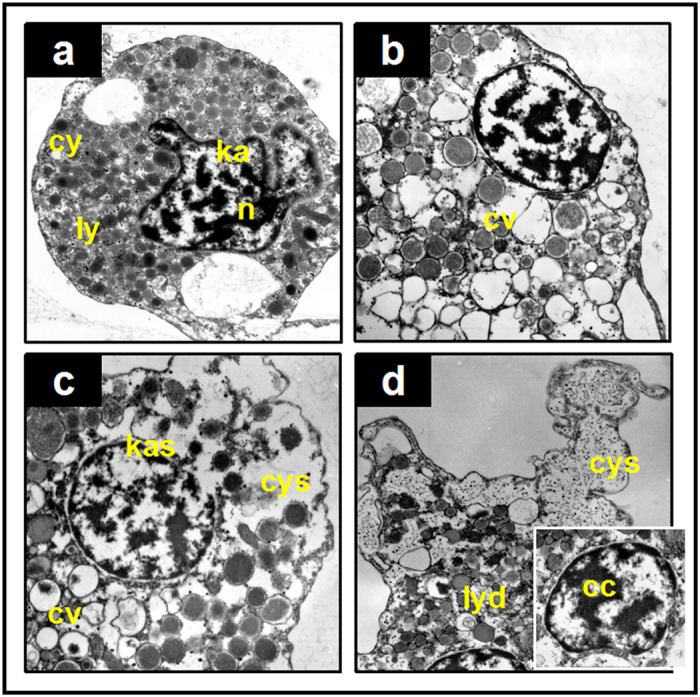
Representative TEM micrographs of observed histopathological alterations in the granulocyte of *M*. *edulis*. (**a**) Normal structure of a granulocyte of a mussel, with dense and boundary clear lysosomes (ly), complete and smooth cytomembranes (cy) and karyothecas (ka), and chromatin evenly distributed in the nucleus (n). (**b**) Cytoplasmic vacuolation (cv), with many lysosomes having lost their contents. (**c**) Swollen cytomembrane (cys) and swollen karyotheca (kas), with obvious membrane separations. Severe swelling causes breakages. (**d**) A seriously injured granulocyte, with a fuzzy boundary of lysosomes (lysosomes dissolved, lyd), chromatin condensation (cc) and extensively swollen cytomembrane (cys).

Although no significant change was observed in the population level, we found clear organelle damage in haemocytes with TEM analysis. Various alterations were observed in granulocytes: cytoplasmic vacuolation (Fig. 1b), cytomembrane and karyotheca swelling (Fig. 1c), and lysosomes dissolution and chromatin condensation (Fig. 1d). The ratio of the damaged cells increased with pH increment compared to the control, but most of the granulocytes were not damaged. We randomly chose approximately 100 cells under microscopic view in each group; the cells possessing at least 2 alterations were counted, and the ratios were calculated as approximately 13% (control), 23% (7.7 HG), 19% (7.7 CG), 29% (7.1 HG) and 35% (7.1 CG) (Table 1). Seawater acidification seemed to be able to damage the sub-cellular structure of the granulocyte, but these impairments seemed to not be serious enough to alter the population level under the present experimental conditions.

We applied the average histopathological indexes (*I*_*h*_) to quantitatively estimate the impairment in each treatment (Table 1). Significantly high *I*_*h*_ values were found in the groups at pH 7.1 (P < 0.05), showing histopathological impairments caused by low pH. We found little difference in the groups at pH 7.7 compared to the control (P > 0.05). In addition, the *I*_*h*_ in CG at pH 7.1 was 1.3 times higher than that in HG at pH 7.1 (p = 0.01, <0.05), indicating more severe damage from the CG treatment. The scores of each alteration (*W*_*j*_* × a*_*jh*_) showed that cytoplasmic vacuolation was a sensitive biomarker for acidification, and the damage to the nucleus from CO_2_ in high concentration was significantly more serious than the damage to the nucleus from HCl (Table 1).

**Figure 2:**
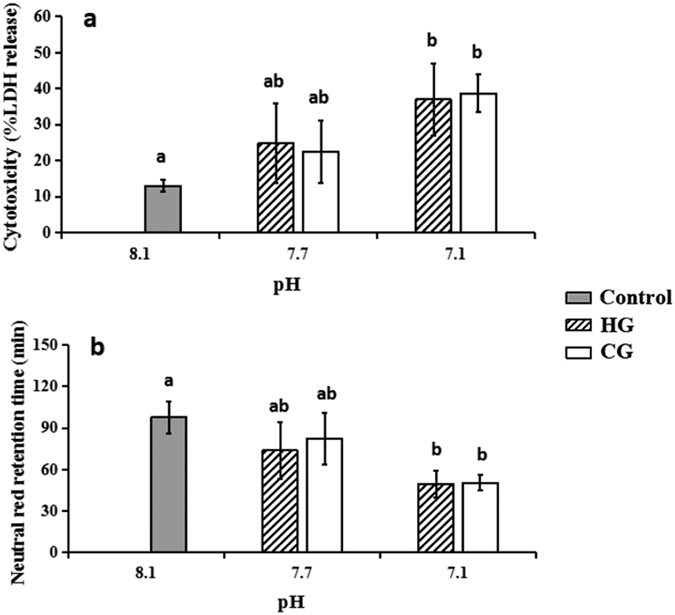
Structural damaging effects of seawater acidification induced structural changes in haemocytes exposed to seawater acidification (mean ± SEM, n = 5). Note: Different lower case letters indicated the significant difference between the treated groups with the control group at the P < 0.05 level. (**a**) Acidification caused membrane damage in haemocytes detected by LDH release assay; (**b**) Acidification caused lysosomal membrane stability damage in haemocytes detected by NRRT assay.

The results of the present study also showed that a lower pH level induced more serious membrane damage, confirming the membrane toxicity of acidification. The LDH release was found to be no more than 15% (13.0 ± 1.7%) in the control group of haemocytes, whereas the release concentrations at pH 7.7 (P = 0.004, <0.05) and 7.1 (P = 0.009, <0.05) increased to approximately 2 and 3 times the release concentration in the control, respectively (Fig. 2a). No substantial difference was found between HG and CG at the same pH level. At the same time, notable changes were detected in the NRRT in each treatment (Fig. 2b). The neutral red was confined to the lysosomal compartment for approximately 60 min in the control group, which was significantly longer than for pH 7.1 in both acidified groups (P = 0.002, <0.05). By contrast, the retention times in both groups at pH 7.7 did not significantly change. In general, the damage to the lysosomal membrane stability in haemocytes caused by HCl addition was similar to the damage caused by CO_2_ enrichment at a consistent pH level.

### Effects of seawater acidification on the haemocyte phagocytosis ability

**Figure 3:**
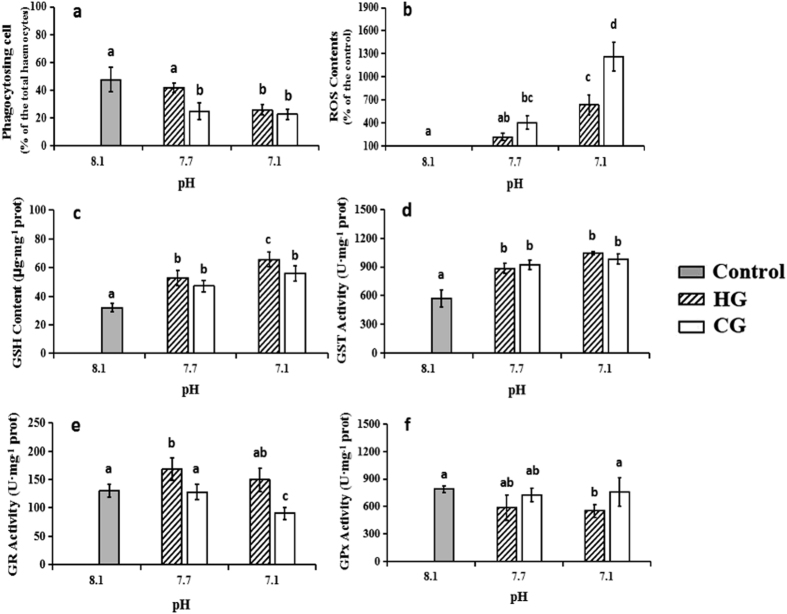
The functional effects of seawater acidification in haemocytes exposed to HG or CG induced seawater acidification at different pH values compared to the control group (mean ± SEM, n = 5). Different lower case letters indicate significant differences between haemocyte treatments (P < 0.05, ANOVA). (**a**) Phagocytosis levels of haemocytes; (**b**) Percent differences in production of ROS in haemocytes; (**c**) The concentration of GSH in haemocytes; (**d**) GST activity changes in haemocytes; (**e**) GR activity changes in haemocytes; (**f**) GPx activity changes in haemocytes.

Exposure to reduced pH decreased the observed phagocytosis levels. The phagocytosis levels decreased with decreasing pH in both groups and eventually decreased to half the control level in the groups at pH 7.1 (P = 0.004 in the HCl group, P = 0.003 in the CO_2_ group, <0.05) (Fig. 3a). The inhibition of phagocytosis seemed to be more sensitive to CO_2_ than HCl at pH 7.7.

### Effects of seawater acidification on ROS production and glutathione-related antioxidant activities

The assay results indicated that the ROS concentration in *M*. *edulis* haemocytes can be activated by acidification (Fig. 3b) and that a lower pH value induced stronger ROS production. There was no significant difference between the two treatment groups at pH 7.7, whereas the ROS content in the CO_2_ group was approximately 1.8 times higher than the ROS content in the HCl group at pH 7.1 (P = 0.001, <0.05).

The effects of HCl and CO_2_ on haemocyte GSH content by acidification are shown in Fig. 3c. A considerable increase in the GSH content was observed in the acidified groups compared to the control subjects. However, the effect was more pronounced in the HCl group than in subjects exposed to CO_2_. The GST activity was increased in the haemocytes of *M*. *edulis* that were exposed to acidification compared to the control (Fig. 3d). The subjects exposed to HCl and CO_2_ did not have significant differences at each pH level. The GR activity in the haemocytes of the seawater acidification cases is presented in Fig. 3e. Subjects exposed to HCl had an increase in the GR activity compared to the control subjects, whereas a decrease was observed in the CO_2_ group. Unlike the GR activity, HCl addition could inhibit the GPx activity, while CO_2_ enrichment had no significant effect (Fig. 3f).

## Discussion

The ability of a bivalve to respond to environmental stress depends to a significant degree on the viability and functional capability of haemocytes^19^. In our study, seawater acidification obviously affected the structure and immune function of the haemocytes in *M*. *edulis*, suggesting that responses to acidifying stress occurred. We applied the two acidifying methods of HCl adjustment and CO_2_ enrichment in this study, and both showed similarly negative effects, indicating that the increasing H^+^ concentration in the culture system was the decisive factor. However, we also found that the haemocytes in the CO_2_-seawater were more vulnerable compared to those in the HCl-seawater at the same pH level, which was presumed to imply that factors other than H^+^ might play an essential role in the negative impacts. The sub-cellular structure of the haemocytes was also observed to change significantly with acidification exposure: the chromatin condensed, the lysosomes dissolved, cytoplasm vacuolation increased, and the cytomembranes and karyothecas became swollen. Since the nucleus plays important roles in all aspects of growth, reproduction, metabolism and protein synthesis^20^, these damages would result in abnormality in *M*. *edulis*. Vacuole changes are considered to relate closely to lysosomal dysfunction^21^. The enlarged vacuole and the decreased NRRT were observed simultaneously in this study, which indicated the involvement of lysosomal damage in the sub-cellular structure changes. In addition, LDH leakage is another important index indicating the cellular membrane stability^22^,^23^, and the acidification-induced LDH leakage changes indicated that the damages to the cellular structure resulted in the cellular function changes. Although we found the sub-cellular impairments, most granulocytes cells were not damaged. The percent of damaged granulocytes was calculated to be no more than 35%, even in the highest treatment groups. Regarding the small difference in population mortality, the possible explanation might be that the sub-cellular damage threatened but was not lethal to the cell growth. Since ecosystems are hierarchical, this small alteration at the sub-cellular level would ultimately create impairments at the population level. We thus speculated that a change in population level might occur in the near future as stressing exposure increases.

The ratio of granulocytes in the haemocyte community and their phagocytosis have been noted as the indicators of immune function in the present study. In this study, phagocytosis was greatly inhibited. Since phagocytosis is generally suggested to be based on the abilities of cytomembrane packaging and lysosomes elimination^24^, the present results confirmed that damages to cytomembrane and lysosomes were responsible for the phagocytosis inhibition. We thus believe that on the premise that elevated H^+^ concentration would not change the average number of haemocytes circulating in mussels^25^,^17^, phagocytosis depression would surely weaken the overall immune function of *M*. *edulis*.

Oxidative stress is suggested to be involved in the toxic mechanism of seawater acidification^26^. Our results demonstrated that acidification exposure elevated the ROS level in haemocytes in a concentration-dependent manner and resulted in the occurrence of oxidative stress. This might be because the low pH negatively affected the efficiency of the mitochondrial electron transport chain (ETC) by increasing the electron slip in the ROS-generating mitochondrial complexes I and III and/or by partially inhibiting the flow through the downstream ETC complexes^27^,^28^. ROS possess strong biological activity and can react with almost all intracellular organic compounds, including DNA, proteins and lipids^29^,^30^,^31^. This reactivity could be used to explain how the acidification damaged cytomembranes, lysosomes and chromatin. ROS also attack recognition receptors on the cytomembrane and reduce the fluidity of cytomembranes by decreasing the unsaturated fatty acid content^22^,^32^, and this is why the inhibition of phagocytosis occurs with acidification exposure in this study. Additionally, Tomanek *et al*.^26^ considered that the cytoskeleton protein was the main target of ROS induced by acidification^26^. Due to the important functions of the cytoskeleton protein in cells^33^, we speculated that ROS-mediated damage to the ultrastructure of haemocytes might be more serious and irreversible than what we had observed.

**Figure 4:**
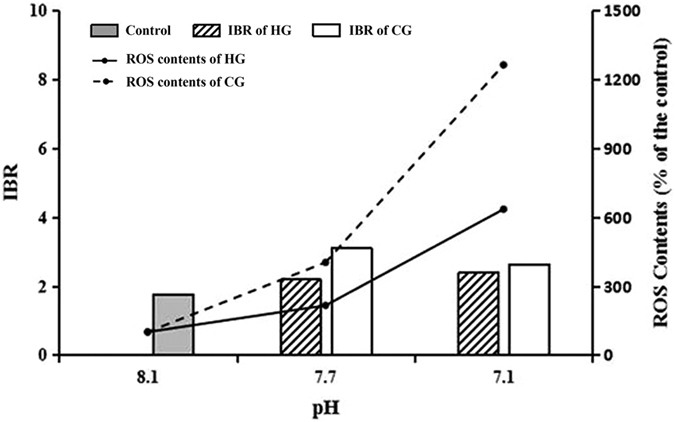
Changes of antioxidant system (IBR consolidation) and ROS concentration in each pH level on 21 d in the HCl adjustment and the CO_2_ enrichment groups .

It is well known that a balance exists between the production and elimination of ROS in organisms under normal circumstances, and the antioxidant system plays an essential role in keeping that balance. However, that balance would be disturbed when exposed to stresses^34^. We found an increase in the ROS level and an alteration of the main components of the antioxidant system with acidification exposure, and the relationship between them was also quantitatively analysed. Four antioxidant system components (GSH, GST, GR, and GPx) were combined into one integrated biomarker response (IBR) index^10^, and we found a positive relationship between IBR and ROS production, which meant that the antioxidant system responded actively to the acidification (Fig. 4). However, compared with pH 7.7, IBR at pH 7.1 did not obviously increase even as ROS production increased significantly, indicating that acidification might inhibit the antioxidant system response. This inhibition was probably caused by the insufficient supply of antioxidant enzymes induced by environmental stress, which enhanced the formation of intracellular ROS^35^.

Although the two methods of acidification presented similarities in immune function inhibition in *M*. *edulis*, it was especially noteworthy that CO_2_-seawater seemed to have more serious effects, especially under mild acidification conditions (pH 7.7). Since the dissolved CO_2_ and HCl had different ions in the culture system, we believed that H^+^ was a decisive but not the only factor that resulted in the negative impact of seawater acidification. Three kinds of changes would occur when CO_2_ gas dissolved into the seawater: increasing H^+^ concentration, breaking of the original carbonate balance, and increasing the molecular form of CO_2_ in the water. It has been proven that uncharged CO_2_ molecules can readily diffuse into the cells through the cytomembrane, according to the *p*CO_2_ gradient^18^,^36^. Bibby *et al*.^17^ and Li *et al*.^15^ had reported that elevated haemolymph Ca^2+^ concentration, which was induced by an increased H^+^ concentration and a changed carbonate balance, was likely to alter the immune functions of haemocytes by disturbing the calcium-dependent signalling in key physiological processes, such as phagocytosis^15^,^17^. In addition, CO_2_ can increase ROS production by directly reacting with peroxynitrite (ONOO^-^), resulting in the formation of reactive carbonate, oxygen and nitrogen species that can oxidize multiple cellular compounds, including thiols, aromatic compounds, cytochromes and other haeme-containing molecules, thus resulting in oxidative stress^37^,^38^,^39^. Therefore, ROS production was higher and phagocytosis was lower in CO_2_-seawater than in HCl-seawater in this study. Another presumption for the explanation might be from the different provision of energy between acidification induced by CO_2_ and HCl. Our previous studies found that the filtering rate of mussels in CG maintained only 47% of HG at pH 7.7^10^, which meant its total energy intake was only 47% of the HG. Andrenodi *et al*. (1990) found that ROS production consumed ATP in the cells^40^. Our newfound results also showed that the intracellular ATP level in haemocytes in CG (0.084 ± 0.006 ng/10^4^ cells) was 76% of the level in HG at pH 7.7 (0.111 ± 0.009 ng/10^4^ cells) (Control: 0.107 ± 0.004 ng/10^4^ cells; 7.1 HG: 0.066 ± 0.004 ng/10^4^ cells; 7.1 CG: 0.056 ± 0.007 ng/10^4^ cells. Sun, unpublished data). To further prove the assumption, we analysed the relationship among the filtering rate, ATP concentration, ROS production and phagocytosis by Pearson’s analysis (Table 2) and found that ROS production and phagocytosis showed, respectively, significantly negative (P < 0.05) and positive (P < 0.05) correlations with ATP concentration in CG, but no significance was observed in HG. We thus speculated that there might be a link between the intracellular energy crisis and immune function inhibition in haemocytes of *M*. *edulis* in CG. However, further research on the energy crisis and the potential link between it and immune function is needed. In addition, we also obtained a good correlation between ROS production and phagocytosis in both CG and HG (Table 2), which demonstrated that the overproduction of ROS might be a possible mechanism to explain the damage to the haemocyte induced by seawater acidification.

**Figure 5:**
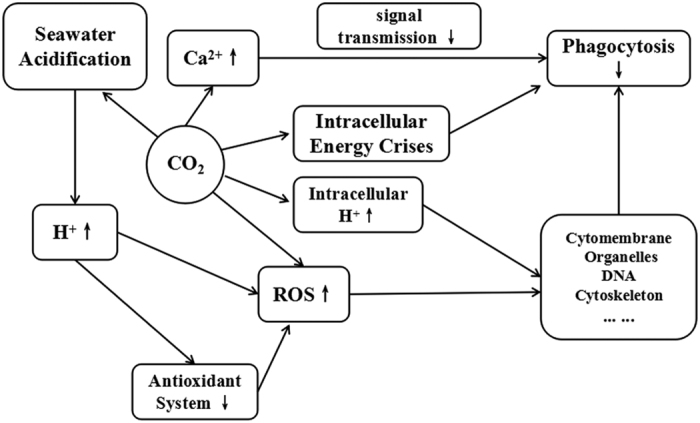
The conjectured pathway of how seawater acidification acts on the structure and immune function of haemocytes of *M*. *edulis* .

This is the first comparative study of changes to the immune function of haemocytes induced by seawater acidification with different treatment methods. When considering the results in the present study, we presumed that seawater acidification might affect the structure and immune function of *M*. *edulis* haemocytes through the following pathway (Fig. 5). Acidification exposure resulted in the overproduction of ROS, which were responsible for inducing oxidative stress in the haemocytes. At the same time, acidification induced further accumulation of ROS by inhibiting the function of the antioxidant system. The excessive ROS accumulation exerted negative effects on the haemocyte ultrastructure. Since the functional performance of the cells was based on their structural integrity, the structural damage to the haemocytes resulted in immune inhibition. In addition to the effects mentioned above, CO_2_-induced seawater acidification might cause an energy crises, increase intracellular Ca^2+^ and H^+^, or even participate directly in the ROS production, which finally worsens the situation. It was shown that CO_2_-induced seawater acidification induces multiple stresses, which were dominated by, but not limited to, the increased H^+^ concentration. Further research should focus on analysing the energy metabolism of *M*. *edulis* exposed to different methods of seawater acidification to elucidate the deep-rooted mechanisms.

## Materials and Methods

### Mussel acclimation and maintenance

The blue mussels, *Mytilus edulis* (shell length 45.65 ± 0.54 mm and weight 6.32 ± 0.75 g), were caught in Laoshan Bay, Qingdao, China (36°15′N and 120°40′E). They were left undisturbed in 200-L aerated natural seawater tanks (pH 8.0 ± 0.1, salinity 31 ± 1.0, and 23 ± 1 °C) for 7 days of acclimation. During the experiment, 30 randomly selected mussels were placed in 15 experimental tanks (vol. = 8 L; 450 mussels in total) that were continuously supplied with seawater from five 100-L header tanks (200 mL/h). Two hundred milligrams (dry mass·tank^−1^·day^−1^) of food algae, *Platymonas helgolandica* (Chlorophyta), was diluted in seawater and supplied to the holding tanks by gravity feed (approximately 1 mL·min^−1^), and the final density in each tank was 1.5 × 10^5^ cells·min^−1^. The experiment lasted for 21 days and was repeated twice.

### Setting up the acidifying system

Two different acidifying methods, CO_2_ enrichment and HCl adjustment, were evaluated in the present study. The test seawater was prepared as follows: (1) CO_2_ group (CG): seawater was bubbled with pure CO_2_ gas (99.9%); (2) HCl group (HG): seawater was acidified by adding 1 M HCl. Three pH levels were evaluated in the present study: pH 8.1 (ambient seawater pH, *p*CO_2_ ≈ 390 ppm), 7.7 (2100, Orr *et al*.^41^; *p*CO_2_ ≈ 1500 ppm; CO_2_ enrichment)^41^, 7.7 (HCl addition), 7.1 (CCS leak; Berge *et al*.^42^; *p*CO_2_ ≈ 5000 ppm; CO_2_ enrichment)^42^, and 7.1 (HCl addition). The pH_NBS_ values of seawater in the header tanks were measured and adjusted by pH controllers (pH/ORP-101, HOTEC, Taiwan; pH fluctuations were controlled within 0.08 units). The salinity was measured daily using a handheld salimeter (WY028Y, HUARUI, CHN). The total alkalinity (A_T_) was measured weekly using an open-cell potentiometric titration technique. All other carbonate system variables were calculated using the CO2SYS software according to the method described by Pierrot *et al*.^43^. Carbonate chemistry data are presented in Table 3.

### Haemocyte extraction

Haemocytes in all treated and control groups were extracted on the 21^st^ day after exposure. The haemocytes were extracted using the description in a patent (No: CN204705520U) established by us. The extracted haemocytes were held on ice to limit spontaneous activation and reduce clumping before use. Haemocytes from 5 mussels in the same group were pooled, and 3 replicates were prepared. The mixed haemocytes were diluted with an equal volume of anticoagulant solution (Alsever’s Solution) and centrifuged at 400× g for 10 min. The haemocyte pellets were resuspended and used for subsequent operations.

### Haemocyte parameter measurements

Haemocyte mortality was measured using PI (propidium iodide) fluorescence with a flow cytometer (Epics XL, BECKMAN COULTER, FL, USA). Haemocytes were fixed in cold 70% ethanol and resuspended in PBS containing 20 μg/mL PI (SIGMA, MO, USA). Cells were processed for FC analyses at 488 nm. On a log scale of FL2, we evaluated the percentage of dead haemocytes relative to the total number of haemocytes.

Because of the important phagocytic activity of the granulocytes, the proportion of granulocytes in the total haemocytes was used to indicate the total phagocytic ability of the haemocytes. Flow cytometric analyses were performed as previously described by Allam *et al*. (2001) using a flow cytometer^44^. Signals were recorded on FS (relative size) × SS (granularity) plots, and discernible groups were gated according to the SS cell peak figures. The ratios of the granulocytes to the whole haemocyte population were calculated. Transmission electron microscopy (TEM, H-7000, HITACHI, JPN) was applied to determine the granulocyte ultrastructure according to Kuchel *et al*.^45^. The haemocyte pellets were embedded in 1.5% w/v low melting agarose (26~30 °C, SIGMA, MO, USA) after centrifugation to avoid damage caused by hyperpyrexia. Sections (15 sections per slide; 3 slides per treatment; approximately 100 granulocytes in total) were observed and recorded. The semiquantitative histopathological indexes for the granulocytes were based on the weighed indexes originally proposed by Costa *et al*.^46^ for clams with modifications^46^,^47^. Briefly, the histopathological assessment considered the concepts of biological significance (weight, see Fig. 1) of the alteration to which a value between 1 (minimal significance) and 4 (maximum severity) was assigned. Therefore, the highest weight (w = 4) was attributed to chromatin condensation, followed by organelle dissolution (w = 3) and swelling of the cytomembrane and karyotheca (w = 2). Cytoplasmic vacuolation was given the lowest weight (w = 1) for having been described as having ‘damage limitation’^47^. The score was a value of 0 (feature/alteration not observed), 1 (visible/local alteration) or 2 (diffuse). The histopathological indices were calculated using Formula (1), as described by Costa *et al*.^46^:


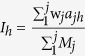


where *I*_*h*_ is the histopathological condition index for the individual h, *w*_*j*_ is the weight of the j^th^ histopathological alteration, *a*_*jh*_ is the score attributed to the h^th^ individual for the j^th^ alteration and *M*_*j*_ is the maximum attributable value for the j^th^ alteration. The denominator of the equation normalizes *I*_*h*_ to a value between 0 and 1, thus permitting comparisons between different conditions, such as different organs, sampling sites or campaigns.

The plasma membrane damage was evaluated by quantifying the lactate dehydrogenase (LDH) release. Half a millilitre of fixed haemocytes (accurate calibration at 2 × 10^5^/mL) was placed in a 1.5 mL centrifuge tube; then, the level of LDH released in the supernatant was detected using an LDH cytotoxicity assay detection kit (BEYOTIME, CHN) according to the manufacturer’s instructions.

The neutral red retention time (NRRT) procedure was performed according to that described in Hauton *et al*.^48^. Briefly, 50 μL of haemocyte suspension (approximately 2 × 10^5^/mL) was pipetted onto an alcohol-cleaned glass slide and incubated for 15 min. A total of 50 μL of the diluted neutral red solution (prepared according to the procedure of Lowe and Pipe, 1994) was added to the slides, and then the samples were covered with a coverslip (20 × 40 mm)^49^. Every 10 min, 3 randomly selected fields of view per slide were examined using bright field (×400 magnification; CX31, OLYMPUS, JPN). The endpoint of the assay was defined as the time at which 50% of the granulocytes lost dye from their lysosomes.

A phagocytosis assay was performed according to the method of Hégaret *et al*.^19^. Haemocytes (accurate calibration at 4 × 10^5^/mL) were diluted with an equal volume of anticoagulant solution. Fluorescent beads (Fluoresbrite YG Microspheres, 1.00 um; Polysciences) were added into the samples at a concentration of 50:1 (beads:haemocytes). Samples were analysed on the flow cytometer (Epics XL, BECKMAN COULTER, FL, USA) after a 60 min incubation at 20 °C. On the FL1 histogram, all haemocytes showing fluorescence were included in a marker for calculating phagocytosis.

ROS production was measured by the oxidation of non-fluorescent DCFH-DA (2′,7′-dichlorofluorescin diacetate) to fluorescent DCF. The haemocyte suspensions in each treatment group were accurately calibrated at 2 × 10^5^/mL. Then, they were labelled with 10 μM DCFH-DA (SIGMA, MO, USA) and incubated in the dark for 30 min at 23 °C. Then, the cells were centrifuged to remove the excess fluorescent probe and resuspended in PBS containing anticoagulant solution. The fluorescence values were detected with a fluorescence microplate reader (Enspire, PerkinElmer, USA). The percentage differences in fluorescence produced by haemocytes in experimental treatments were compared to control haemocytes.

The following four antioxidant system components, which are closely related to the glutathione detoxification cycle, were selected for activity analysis: glutathione peroxidase (GPx), glutathione S-transferase (GST), glutathione reductase (GR) and glutathione-SH (GSH). Haemocytes (approximately 2 × 10^5^/mL) were destroyed using an ultrasonic wave (ice-cold) and centrifuged. The supernatant was used for enzymatic analysis via spectrophotometry (UV-8000, METASH, CHN). The GPx activity was measured using the decrease in NADPH at 340 nm according to the method described by Livingstone *et al*.^50^. The GST activity against CDNB (1-chloro-2,4-dinitrobenzene) was determined at 340 nm as described by Habig *et al*.^51^. The GR and GSH activity levels were measured in accordance with the methods described by Foyer and Halliwell (1976) and Griffith (1980)^52^,^53^, respectively. The protein content was measured according to the procedure of Bradford^54^.

### Statistical analyses

The mean values and standard errors were calculated from different replicates of each treatment (n = 5), and the figures were generated using the Sigmaplot 12.5 software. The differences between the treated groups and controls were analysed by one-way ANOVA Student-Newman-Keuls using SPSS 22.0 software, and significance was set to P < 0.05.

## Additional Information

**How to cite this article**: Sun, T. *et al*. Seawater acidification induced immune function changes of haemocytes in *Mytilus edulis:* a comparative study of CO_2_ and HCl enrichment. *Sci. Rep.* **7**, 41488; doi: 10.1038/srep41488 (2017).

**Publisher's note:** Springer Nature remains neutral with regard to jurisdictional claims in published maps and institutional affiliations.

## Figures and Tables

**Table 1:** Respective alteration weights, scores of each alteration (*W*_*j*_ × *a*_*jh*_) and average granulocyte histopathological indexes (*I*_*h*_, ± 95% confidence intervals) for each treatment group.

Alteration	Weight	*W*_*j*_ × *a_jh_*				
		C	7.7 HG	7.7 CG	7.1 HG	7.1 CG
Cytoplasmic vacuolation	1	0.3 ± 0.12^a^	0.9 ± 0.21^b^	0.8 ± 0.36^b^	1.0 ± 0.31^b^	1.2 ± 0.18^b^
Swollen cytomembrane	2	0.2 ± 0.10^a^	0.5 ± 0.23^a^	0.4 ± 0.12^a^	1.1 ± 0.35^b^	1.5 ± 0.40^b^
Swollen karyotheca	2	0.7 ± 0.06^a^	0.9 ± 0.35^a^	0.8 ± 0.25^a^	0.9 ± 0.27^a^	1.4 ± 0.33^a^
Lysosomes dissolved	3	0.3 ± 0.15^a^	1.0 ± 0.64^a^	0.7 ± 0.11^a^	2.5 ± 0.59^b^	3.1 ± 0.48^b^
Chromatin condensation	4	0.1 ± 0.04^a^	0.1 ± 0.06^a^	0.1 ± 0.02^a^	0.3 ± 0.14^b^	0.6 ± 0.17^c^
*I_h_*		0.07 ± 0.02^a^	0.14 ± 0.03^a^	0.12 ± 0.02^a^	0.25 ± 0.03^b^	0.33 ± 0.05^c^
Alteration more than 2 indexes (%)		13 ± 3.8^a^	23 ± 4.9^a,b^	19 ± 8.4^a,b^	29 ± 4.0^b,c^	35 ± 4.5^c^

Different letters indicate significant differences within each reaction pattern (P < 0.05). C: control; 7.7 HG: HG at pH 7.7; 7.7 CG: CG at pH 7.7; 7.1 HG: HG at pH 7.1; 7.1 CG: CG at pH 7.1.

**Table 2:** Pearson’s correlation coefficients for the filtering rate, ATP concentration, ROS production and phagocytosis of *M*. *edulis*.

	Filtering Rate	ATP Concentration	ROS Production
**HG**			
Filtering Rate _(Sun *et al*., 2016)_			
ATP Concentration _(Sun, unpublished)_	0.406		
ROS Production	−0.880**	−0.58	
Phagocytosis	0.679*	0.394	−0.873**
**CG**			
Filtering Rate _(Sun *et al*., 2016)_			
ATP Concentration _(Sun, unpublished)_	0.606		
ROS Production	−0.712*	−0.771*	
Phagocytosis	0.667*	0.732*	−0.689*

*Significant; **Extremely significant.

**Table 3:** Physicochemical parameters in the carbonate system for each condition in the CO_2_ groups.

pH_(setting)_	pH_(measured)_	TA_(mM)_	*p*CO_2(μatm)_	H_2_CO_3(mM)_	HCO_3_^−^_(mM)_	CO_3_^2−^_(mM)_	ΩAg	Ω_Cal_
8.1	8.12	2020	408.61	13.20	1628.18	156.87	2.44	3.75
7.7	7.66	2016	1349.55	43.10	1875.68	56.93	0.88	1.36
7.1	7.07	2022	5483.50	177.10	1980.95	15.45	0.24	0.37

Measured parameters pH and determined total alkalinity (At) from weekly water samples. Partial CO_2_ pressure (*p*CO_2_), carbonic acid (H_2_CO_3_), bicarbonate (HCO_3_^−^) and carbonate ion concentrations (CO_3_^2 −^); calculated (CO_2_SYS software, Pierrot *et al*.^43^) saturation states of aragonite (ΩAg) and calcite (Ω_Cal_).
